# Polygenic study of endurance-associated genetic markers *ACE I/D*, *ACTN3 Arg(R)577Ter(X)*, *CKMM A/G NcoI* and *eNOS Glu(G)298Asp(T)* in male Gorkha soldiers

**DOI:** 10.1186/s40798-017-0085-0

**Published:** 2017-04-26

**Authors:** Seema Malhotra, Kiran Preet, Arvind Tomar, Shweta Rawat, Sayar Singh, Inderjeet Singh, L. Robert Varte, Tirthankar Chatterjee, M. S. Pal, Soma Sarkar

**Affiliations:** 1 Defence Institute of Physiology and Allied Sciences (DIPAS), Ministry of Defence. Government of India, Lucknow Road, Delhi, 110054 India; 2 Defence Research and Development Establishment (DRDE). Ministry of Defence, Government of India, Jhansi Road, Gwalior, 474002 Madhya Pradesh India

**Keywords:** Polygenic, Endurance, Gorkha, Indian, *ACE*, *ACTN3*, *CKMM*, *eNOS*

## Abstract

**Background:**

Gorkhas, a sub-mountainous population of the Himalayan region, are known for strength and bravery. In the present study when “Gorkha” is used without brackets, we are mentioning Gorkhas of Tibeto-Burman origin. Physical capability, strength and endurance are important components of fitness associated with genetic traits. The aim of this study was to examine the endurance potential of male Gorkha soldiers, based on endurance-related genetic markers *ACE I/D*, *ACTN3 Arg (R)577Ter(X)*, *CKMM A/G NcoI* and *eNOS Glu(G)298Asp(T)*.

**Methods:**

Genotypic and allelic frequencies were determined in 374 male Gorkha soldiers (Tibeto-Burman). These frequencies were compared with frequencies obtained from Gorkha (Indo-Aryan), high-altitude natives (Tibeto-Burman) and Indian lowlanders (Indo-Aryan). “Total genotype score” (TGS) was calculated from accumulated combination of polymorphisms with maximum value “100” for theoretically “optimal” polygenic score. Probability of occurrence of “optimal” endurance profile was also determined.

**Results:**

*ACE II* genotypic frequency was highest in Tamangs followed by Gurungs, Rais, Limbus and Magars. No statistical difference in genotypic and allelic frequency of *ACTN3 Arg(R)577Ter(X)* was noted within the groups. Rais showed the highest *CKMM A* allele frequency (0.908) compared to other Gorkha (Tibeto-Burman) groups. Limbus and Tamangs showed the highest *eNOS G* allele frequency (0.938 and 0.915, respectively) compared to that of other groups. Probability of male Gorkha soldiers possessing a theoretically optimal polygenic endurance profile for four candidate polymorphisms was ~3.35% (1 in 30). Four percent of the population of male Gorkha soldiers (15 in 374) exhibited an optimal TGS 100, and 16% exhibited TGS 87 for endurance compared to male Indian soldiers belonging to the lowland (Indo-Aryan) and Gorkha (Indo-Aryan) populations suggesting an overall more “favourable” polygenic profile in the male Gorkha soldier (Tibeto-Burman) population.

**Conclusions:**

This study presents evidence of higher frequency of endurance-associated genes in the Gorkhas implying thereby that such genetically endowed individuals from the population may be selected and trained for achieving excellence in endurance-related elite sports activities.

**Electronic supplementary material:**

The online version of this article (doi:10.1186/s40798-017-0085-0) contains supplementary material, which is available to authorized users.

## Key points

- This study was conducted on male Gorkha soldiers of the Tibeto-Burman linguistic phyla wherein genetic studies are limited. The study reports for the first time the genotypic and allelic frequencies of four endurance-associated genetic markers in the male Gorkha soldiers.
- The study shows that nearly 4% of the male Gorkha soldiers exhibit an “optimal” total genotypic score (100) for endurance based on four polymorphisms, indicating the genetic potential of this population for achieving excellence in endurance-related elite sports activities.

## Background

*Gorkhas* (also spelled as *Gurkhas* 1) are a sub-mountainous population of the Himalayan region (Nepal) and make excellent soldiers. During the Anglo-Nepalese war of 1814–1816, the British were greatly impressed by the bravery of the Nepalese soldiers and started recruiting Nepalese to the Gurkha regiments of the British Indian Army [1]. The soldiers in the British army were mainly recruited from the “true Gorkha martial tribes” of *Gurung*, *Magar*, *Rai*, *Limbu*, *Thakur*, *Chhetri* and *Sunawar* [2]; the Indian Army continues to recruit from the same brigade of Gorkhas. *Gurungs*, *Magars*, *Rais*, *Limbus*, *Tamangs* and *Sherpas* are associated with Tibeto-Burmese cultural traditions and physical features conventionally labelled as Mongoloid while *Thakur* (*Brahmin*) and *Chhetri* castes are associated with Aryan cultural traditions and have physical features conventionally labelled as Caucasoid [3]. Gurkha Service opened an opportunity for the soldiers to settle in different parts of the British Empire including India, and their descendants are present in Assam, Sikkim, Darjeeling and Dehradun [3].

The Gorkha soldiers are best known for their physical strength, fighting tenacity, bravery and fearlessness in battle [1, 4]. Physical capability, strength and power are important components of fitness. Himalayan Sherpas are well known for their physical strength and endurance in the high-altitude terrain. Himalayan Sherpa elite climbers demonstrated high functional reserve with maximal oxygen uptake (VO_2max_) of 66.7 ± 3.7 ml min^−1^ kg^−1^, maximal cardiac frequency of 199 ± 7 beats min^−1^ and ventilatory anaerobic threshold of 62 ± 4% of VO_2max_ [5]. Higher frequency of *I* allele of *ACE* (angiotensin-converting enzyme gene, location: 17q23.3) have been reported in Sherpas [6]. *ACE I* allele is associated with endurance-related events [7, 8] and exercise performance in atmospheric hypoxia [9]. Predominance of *ACE II* genotype and *I* allele was demonstrated in male Gorkha soldiers [10]. Many other polymorphisms are associated with endurance-related performance [11]. *Arg(R)577Ter(X)* polymorphism of *ACTN3* (alpha actinin 3 gene, location: 11q 13.1) (functional *R* allele and non-functional *X* allele) is associated with generation of rapid forceful contractions [12] and muscle performance [13] with frequency of *XX*-null genotype (loss of alpha actinin 3) being higher in endurance athletes [14, 15]. In an earlier study, *XX* genotype of *ACTN3 Arg(R)577Ter(X)* polymorphism was observed to be present in 23% of Gorkhas [10]. The *A/G NcoI* polymorphism of *CKMM* (muscle-specific creatine kinase gene, location: 19q13.32) is associated with energy-buffering in the skeletal muscle fibres along with tolerance to skeletal muscle damage [16]. *A* allele and *AA* genotype were significantly higher in endurance athletes and were associated with high values of VO_2max_ [17]. Physical fitness test scores in military recruits were also associated with *A* allele [18]. *eNOS Glu(G)298Arg(T)* (endothelial nitric oxide gene, location: 7q36) polymorphism is linked with endurance performance and endurance elite status [19, 20]. *eNOS* encodes the rate-limiting enzyme for nitric oxide (NO) products [21]. Higher frequency of wild *G* allele of *eNOS Glu298Arg (G894T)* polymorphism was reported in high-altitude natives from Ladakh suggesting advantageous consequences in high-altitude environment [22, 23].

High functional reserve, physical strength and endurance in the hostile high altitudes coupled with higher frequency distribution of some of the endurance-related performance enhancing genetic markers in the mountain population indicates possibility of the mountain population being genetically endowed for endurance- related activities. This natural endowment would set the stage for investigation of prospects of the mountain people for distinctive performance in endurance-related elite sports activities. In the present study, we chose to investigate in male Gorkha soldiers, (i) the genotypic and allelic frequency distribution of four genetic variants associated with endurance performance: *ACE I/D (rs4646994)*, *ACTN3 Arg(R)577Ter(X) (rs1815739 C/T)*, *CKMM A/G NcoI (rs8111989 T/C)* and *eNOS Glu298Asp (rs1799983 G/T)*; (ii) determine the probability for the occurrence of an “optimal” polygenic endurance profile using the four polymorphisms in the population and assess whether individuals were likely to exist who harboured “preferable” genotypes for endurance; and (iii) generate a “total genotype score” (TGS) [11] for finding a likely distribution of genetic endurance potential of the male Gorkha soldiers and compare with virtual data of other populations of male Indian soldiers.

## Methods

### Study population and samples

- Three hundred ninety-four healthy male Gorkha individuals serving in the Gorkha regiment of Indian Army, mean age 29 ± 8 years, participated in the study. The clan ties were determined based on self-report, and ethnic backgrounds were ascertained through ethno-linguistic questionnaire. Classification of linguistic phyla and clusters was as per van Driem [24]. For ascertaining that the individuals were unmixed, both parents had to belong to the same group. Based on the ethno-linguistic questionnaire, the male Gorkha soldier participants belonged to five ethnic groups of Tibeto-Burman linguistic phyla: *Gurungs* (28 participants were from Nepal and 47 were from India), *Magars* (25 participants were from Nepal and 101 were from India), *Rais* (37 participants were from Nepal and 37 were from India), *Tamangs* (36 participants were from Nepal and 18 were from India) and *Limbus* (25 participants were from Nepal and 40 were from India) (Table 1).

**Table 1:** Details of participants based on ethno-linguistic grouping (*n* = 394)

Group	Region of origin	Linguistic phylum and linguistic cluster^a^	*n*, males
Gurung	Nepal	TB, Tamangic	28
Gurung	India		47
Magar	Nepal	TB, Magaric	25
Magar	India		101
Rai	Nepal	TB, Kirantic	37
Rai	India		37
Tamang	Nepal	TB, Tamangic	36
Tamang	India		18
Limbu	Nepal	TB, Kirantic	25
Limbu	India		40

Note: Of the 394 participants, 20 individuals were hypertensive and excluded from subsequent studies. The participants were two *Gurungs* (one from Nepal and one from India), three *Magars* (two from Nepal and one from India), nine *Rais* (six from Nepal and three from India), one *Tamang* (from Nepal) and five *Limbus* (three from Nepal and one from India)

*TB* Tibeto-Burman

^a^Classification according to van Driem [24]
- Repository DNA samples from (i) Gorkhas belonging to the Indo-Aryan linguistic phylum, (ii) Indian lowlanders belonging to the Indo-Aryan linguistic phylum and (iii) high-altitude natives of Ladakh (elevation ~3500 m) belonging to Tibeto-Burman linguistic phylum were used for generating genotypic and allelic frequencies for comparison with male Gorkha soldiers (Tibeto-Burman). The repository DNA samples were from healthy male soldiers of Indian Army.

### Physiological measurements

Body weight, height, and body mass index (BMI), heart rate, systolic and diastolic blood pressures were measured prior to blood draw. Height was measured with an anthropometer (GPM, Switzerland). Body weight and body mass index was measured by Body Composition Analyser (Tanita, Korea). Resting heart rate, resting systolic (SBP) and diastolic (DBP) blood pressures were measured with digital blood pressure monitor (M2 Model, Omron Health Care Company Limited, Japan). Hypertension was defined as BP ≥140/90 mmHg. Out of 394 individuals who initially participated (Table 1), 20 individuals were found to be hypertensive and were excluded from all further studies. The rest of the participants (*n* = 374) were normotensive and not on any medications. Of the normotensive participants, 122 volunteers consented for maximal oxygen uptake (VO_2max_). VO_2max_was assessed on a bicycle ergometer (Ergoline Gmbh, Lindenstr, Germany) using published protocol [25]. Briefly, the initial workload was 50 W, the increment was 25 W · min^−1^, and the target cadence was 60–70 rpm. Subjects kept the pedal rotational speed between 60–65 rpm throughout the test. After warm up for 2 min at 50 W, increments of 25 W were made every minute till exhaustion. The test was completed within 10–12 min including the warm up with maximum load of 200–225 W at 60 rpm. During the test, oxygen consumption (VO_2_), carbon dioxide production (VCO_2_) and heart rate (HR) were continuously recorded using a portable breath by breath gas analysis system (K4b^2^, Cosmed Srl, Italy). Criteria for reaching VO_2max_ were assessed as reaching a plateau despite the increase in work rate or a respiratory exchange ratio value >1.1 or heart rate (HR) reaching >90% of age-predicted maximum HR. All subjects showed at least one of the above-mentioned criteria.

### Genotyping

Venous blood samples (2–3 ml) were obtained at rest in EDTA anticoagulant vacutainers (Beckton Dickinson, CA, USA) and stored at −20 °C till further processing. Genomic DNA samples had A_260_/_280_ ratio of 1.8–1.9 and were adjusted to 20 ng/μl. ~100 to 150 ng of genomic DNA was used for polymerase chain reaction (PCR) amplification in a total volume of 25 μl. Gene variants studied for endurance status are shown in Table 2. Primer sequences and detection of polymorphism in *ACTN3 Arg(R)577Ter(X) (rs1815739)*, *ACE Ins/Del* (*rs4646994*) and *eNOS Glu298Asp* (*rs1799983*) were performed as per published protocol [10, 23], and detection of polymorphism in *CKMM A/G* (*rs1803285*) was performed as per Rivera and co-workers [16]. PCR reaction was run on Gene Amp PCR system 9700 (Applied Biosystem). Samples found to have *ACE DD*and *ID* genotypes were reconfirmed by a second, independent PCR amplification having an insertion-specific sequence [26]. Genotypic profiling for *ACTN3 R577X, CKMM A/G NcoI* and *eNOS Glu298Asp* was performed on repository DNA samples of Gorkhas (Indo-Aryan), Indian lowlanders (Indo-Aryan) and high-altitude natives of Ladakh (Tibeto-Burman). The experiments were conducted in accordance with the quality control measures at the Department of Molecular Biology, Defence Institute of Physiology and Allied Sciences, Delhi, which is an accredited laboratory (ISO 9001:2008).

**Table 2:** Gene variants studied for endurance status

Gene	Location	Polymorphism	Endurance-related marker	Minor allele frequency (MAF) (1000 Genome﻿s)	Ancestral allele
*ACE*	17q23.3	*Alu I/D* *(rs1799752)*	*I*	NA	NA
*ACTN3*	11q13.1	*Arg(R)577Ter (X)* *(rs1815739 C/T)*	*577Ter (X)*	*T* = 0.4008	C
*CKMM*	19q13.32	*A/G NcoI* *(rs8111989 T/C)*	*A*	*C* = 0.3403	T
*eNOS*	7q36	*Glu 298Asp* *(rs1799983 G/T)*	*Glu298(G)*	*T* = 0.1763	G

### Statistical analysis

Allele frequencies and genotype frequencies were calculated by allele counting and analysed by Pearson chi-square (*χ* ^2^) and Fisher’s exact test, respectively. Deviations from the Hardy-Weinberg equilibrium (HWE) were tested for the polymorphisms by comparing observed and expected genotype frequencies with an exact goodness of fit test. For comparison of genotype frequencies within Gorkha sub groups, statistical significance was accepted at *p* < 0.0025 after adjustment with Bonferroni’s correction (alpha = 0.05/20). For inter population comparisons, statistical significance was accepted at *p* < 0.05.

Comparison of maximal oxygen uptake (VO_2max_) between the groups and association of VO_2max_ with genotypes and TGS was performed by one-way analysis of variance (ANOVA) using the software SPSS (Statistical Package for Social Sciences, version 17.0 for Windows; SPSS Inc., Chicago, IL, USA).

### Probability of “optimal” polygenic profile for endurance in the Gorkha soldiers (Tibeto-Burman)

Probability of any given individual possessing the “optimal” genotype from one up to all four polymorphisms, ranked on official gene symbols placed in an alphabetical order, was calculated by using the typical frequency distribution of the genotypes. Based on the typical frequencies of the “optimal” genotypes in male Gorkha soldiers (Tibeto-Burman) and Indian lowlander soldiers (Indo-Aryan), a scale was generated indicating the probability of possessing the “optimal” genetic profile which was then applied to the population. The genetic potential for endurance phenotype of the population was computed by using the algorithm proposed by Williams and Folland [11], and a total genotype score (TGS) was produced. TGS is a bioinformatic approach using predictive algorithm “genotype score” for finding the probability of individuals carrying the preferable genotype for each polymorphism linked to a phenotype [27]. TGS modelling approach uses a simple algorithm resulting from the best accumulated combination of candidate gene polymorphisms. We chose this approach to provide a quantitative way of combining genotype data to predict a complex phenotype. Each genotype within each polymorphism was scored and a combined influence of polymorphisms was computed. A genotype score (GS) of 2 was assigned to the “optimal” homozygous genotype for endurance. A genotypic score of 0 was assigned to “less favourable” homozygous genotype for endurance while the heterozygous genotype received a score of 1. The genotype scores of each single genotype (GS_*ACE*_ + GS_*ACTN3*_ + GS_*CKMM*_ + GS_*eNOS*_) were added up and a TGS was produced as follows:

$$ \mathrm{T}\mathrm{G}\mathrm{S} = \left(100/2\mathrm{n}\right)\times \left({\mathrm{GS}}_{ACE} + {\mathrm{GS}}_{ACTN3} + {\mathrm{GS}}_{CKMM} + {\mathrm{GS}}_{eNOS}\right) $$

where *n* is the number of polymorphisms studied. As suggested by Williams and Folland [11], a TGS of 100 represents the “perfect” polygenic profile of endurance (all GS are 2) and a TGS of 0 represents the “not perfect” profile of endurance. Frequency distribution of the “optimal” endurance genotype in the Gorkha soldiers (Tibeto-Burman) obtained in the present study was compared with the frequency distribution of the optimal endurance genotype in other populations viz., Gorkhas (Indo-Aryan), high-altitude natives (HAN) from Ladakh (Tibeto-Burman), Indian lowlanders (Indo-Aryan), Indian Gujaratis from Houston (Indo-Aryan), HAN Chinese Beijing (Sino-Tibetan), Japanese from Tokyo and Caucasian (European ancestry) taken from published literature and databases.

To examine the distribution of TGS in the Gorkhas (Tibeto-Burman), we created a dataset of 100,000 virtual Gorkha (Tibeto-Burman) individuals from genotypic frequencies of the four polymorphisms obtained in the present study with randomly generated genotypes computed by online software GEMINI [28]. Similarly, a hypothetical data of 100,000 virtual individuals in each population of Gorkhas (Indo-Aryan), Indian lowlanders (Indo-Aryan) and high-altitude natives (Tibeto-Burman) was computed with randomly generated genotypes based on the population-specific genotype frequencies. Distribution of TGS within these virtual populations was examined, and mean and kurtosis statistics were calculated using SPSS, v 17.0 for Windows. The TGS of simulated Gorkha (Tibeto-Burman) population was then compared to the average genotype score of the three hypothetical population datasets.

## Results

### Physiological characteristics

Of the 394 individuals, 151 were from Nepal and 243 were from India. The participants belonged to *Gurung*, *Magar*, *Rai*, *Tamang* and *Limbu* groups of Tibeto-Burman linguistic phyla (Table 1). Twenty individuals (3.26%) were found to be hypertensive [systolic blood pressure (SBP), 152.65 ± 10.08 mmHg; diastolic blood pressure (DBP), 98.25 ± 6.67 mmHg] and were excluded. Within the remaining population, systolic blood pressure and heart rate were observed to be statistically similar (Table 3). Body mass index and diastolic blood pressure (DBP) were significantly different within the population with Rais and Limbus showing the highest DBP (Table 3). Statistically significant difference in VO_2max_ was noted between the groups (Table 4). VO_2max_ was highest in Tamangs (55.80 ± 8.27) followed by Limbus (55.34 ± 6.26) (Table 4, Additional file 1: Table S1).

**Table 3:** Physiological characteristics of the male Gorkha soldiers (TB) (*n* = 374)

Characteristics	Overall	Gurung	Magar	Rai	Tamang	Limbu	*p*
	(*n* = 374)	(*n* = 73)	(*n* = 123)	(*n* = 60)	(*n* = 53)	(*n* = 65)	
Body weight (Kg)	64.51 ± 7.53	65.64 ± 6.8	63.43 ± 6.64	65.89 ± 7.95	63.19 ± 7.69	65.10 ± 9	0.057
Height (cm)	165.43 ± 5.01	165.65 ± 5.84	165.49 ± 4.91	164.24 ± 05.32	166.02 ± 4.29	165.73 ± 4.38	0.390
BMI (Kg/m^2^)	23.49 ± 2.99	23.94 ± 3.59	23.00 ± 3.15	24.34 ± 2.28	22.9 ± 2.52	23.64 ± 2.69	*0.000*
SBP (mmHg)	121.10 ± 12.09	125.21 ± 11.75	121.91 ± 11.57	121.03 ± 13.02	117.80 ± 11.03	119.92 ± 12.27	*0.018*
DBP (mmHg)	73.79 ± 9.99	71.57 ± 11.5	71.16 ± 10.24	77.23 ± 10	72.30 ± 8.36	76.23 ± 8.02	*0.002*
HR (rate/min)	69.35 ± 12.37	69.75 ± 12.88	68.20 ± 11.5	71.10 ± 15.34	66.75 ± 11.11	70.85 ± 9.43	0.894

Values are mean ± SD, significant value <0.05. The significant *p* values are italicized

The values are from participants who were normotensive

*BMI* body mass index, *SBP* systolic blood pressure, *DBP* diastolic blood pressure, *HR* heart rate, *TB* Tibeto-Burman

**Table 4:** Comparison of VO_2max_ between Gorkha groups

	Group ID	Group	VO_2max_ (ml kg^−1^ min^−1^)	Group comparison	Level of significance (*p*)
Overall	–	–	50.82 ± 7.92		
	1	Gurung (31)	46.56 ± 8.18	1 vs 2	ns
	2	Magar (32)	47.97 ± 6.08	1 vs 3	*0.013*
	3	Rai (23)	52.86 ± 6.19	1 vs 4	*0.000*
	4	Tamang (18)	55.80 ± 8.27	1 vs 5	*0.000*
	5	Limbu (18)	55.34 ± 6.26	2 vs 3	ns
				2 vs 4	ns
				2 vs 5	*0.005*
				3 vs 4	ns
				3 vs 5	ns
				4 vs 5	ns

VO_2max_ values are mean ± SD. Level of significance calculated by one-way ANOVA with Tukey’s post-hoc test. VO_2max_ values are from normotensive participants. Number in parenthesis indicates the number of participants in each group

*p* value <0.05 significant. The significant *p* values are italicized

*ns* not significant

### Genotypic and allelic frequency distribution of the studied polymorphisms

Overall genotypic frequency of the studied polymorphism in the male Gorkha soldiers is shown in Table 5. Distribution of homozygous *II* genotype was highest in Tamangs followed by Rais, Gurungs, Limbus and Magars; although, the difference was not statistically significant within the subgroups after Bonferroni correction (*p* < 0.0025). Genotypic frequency of *ACTN3 Arg(R)577Ter(X)* was statistically similar across the Gorkha subpopulation. Both *ACE Ins/Del* and *ACTN3 Arg(R)577Ter(X)* polymorphisms were in Hardy-Weinberg Equilibrium (HWE). Statistical difference was noted in allele frequency distribution of *ACTN3 Arg(R)577Ter(X)* between Gorkhas (Tibeto-Burman), Gorkhas (Indo-Aryan) and high-altitude natives (*p* < 0.05) (Table 6).

**Table 5:** Frequency distribution of the polymorphisms in the male Gorkha soldiers (Tibeto-Burman)

SNP	Variants		Overall	(1) Gurung	(2) Magar	(3) Rai	(4) Tamang	(5) Limbu	Comparison of groups	Genotypic^a^ frequency	Allele^b^ frequency
			Frequency						Level of significance (*p*)		
*rs1799752*	*ACE I/D*	Genotype									
		*II*	0.427	0.465	0.349	0.466	0.509	0.430	1 vs 2	0.14	0.04
		*ID*	0.467	0.438	0.471	0.466	0.452	0.507	1 vs 3	0.81	0.37
		*DD*	0.104	0.095	0.178	0.066	0.037	0.061	1 vs 4	0.45	0.38
		Allele							1 vs 5	0.62	0.99
		*I*	0.671	0.684	0.585	0.700	0.735	0.684	2 vs 3	0.08	0.03
		*D*	0.338	0.315	0.414	0.300	0.264	0.315	4 vs 2	0.02	0.007
									2 vs 5	0.07	0.05
		HWE *p*	0.381	0.893	0.751	0.389	0.230	0.156	3 vs 4	0.75	0.55
									3 vs 5	0.90	0.79
									4 vs 5	0.63	0.38
*rs1815739* *C/T*	*ACTN3* *Arg(R)577Ter(X)*	Genotype									
		*RR*	0.366	0.397	0.308	0.366	0.396	0.413	1 vs 2	0.33	0.13
		*RX*	0.478	0.465	0.487	0.466	0.471	0.492	1 vs 3	0.87	0.61
		*XX*	0.155	0.139	0.203	0.166	0.132	0.092	1 vs 4	0.99	0.97
		Allele							1 vs 5	0.71	0.58
		*R*	0.605	0.630	0.552	0.600	0.632	0.661	2 vs 3	0.69	0.39
		*X*	0.394	0.369	0.447	0.400	0.367	0.338	4 vs 2	0.38	0.16
									2 vs 5	0.10	0.04
		HWE *p*	0.970	0.99	0.882	0.829	0.917	0.423	3 vs 4	0.86	0.62
									3 vs 5	0.45	0.31
									4 vs 5	0.79	0.63
*rs8111989* *T/C*	*CKMM* *A/G NcoI*	Genotype									
		*AA*	0.655	0.630	0.634	0.833	0.603	0.600	1 vs 2	0.89	0.86
		*AG*	0.296	0.287	0.300	0.150	0.377	0.369	1 vs 3	0.02	0.003
		*GG*	0.048	0.082	0.065	0.016	0.018	0.030	1 vs 4	0.22	0.72
		Allele							1 vs 5	0.31	0.83
		*A*	0.803	0.773	0.784	0.908	0.792	0.784	2 vs 3	0.02	0.003
		*G*	0.196	0.226	0.215	0.091	0.207	0.215	4 vs 2	0.32	0.86
									2 vs 5	0.44	0.99
		HWE *p*	0.244	0.280	0.221	0.442	0.283	0.456	3 vs 4	0.02	0.01
									3 vs 5	0.01	0.007
									4 vs 5	0.91	0.88
*rs1799983* *G/T*	*eNOS* *Glu298Asp*	Genotype									
		*GG*	0.799	0.808	0.756	0.766	0.830	0.876	1 vs 2	0.59	0.30
		*GT*	0.184	0.178	0.211	0.216	0.169	0.123	1 vs 3	0.84	0.37
		*TT*	0.016	0.013	0.032	0.016	0.000	0.000	1 vs 4	0.68	0.63
		Allele							1 vs 5	0.41	0.21
		*G*	0.892	0.897	0.862	0.775	0.915	0.938	2 vs 3	0.82	0.98
		*T*	0.108	0.103	0.138	0.125	0.085	0.062	4 vs 2	0.31	0.16
									2 vs 5	0.09	0.02
		HWE *p*	0.387	0.770	0.211	0.941	0.499	0.597	3 vs 4	0.51	0.21
									3 vs 5	0.20	0.04
									4 vs 5	0.47	0.48

Significance is assumed when *p* ≤ 0.0025 after Bonferroni’s correction (alpha = 0.05/20, for four polymorphisms × five ethnic groups)

*HWE* Hardy -Weinberg Equilibrium

^a^Pearson values from chi-square test for genotypic frequency [http://www.physics.csbsju.edu/stats/contingency_NROW_NCOLUMN_form.html]

^b^ *p* values from Fisher’s exact test for allelic frequency [http://www.quantitativeskills.com/sisa/statistics/fisher.htm]

**Table 6:** Comparison of genotypic and allelic frequencies of studied polymorphisms in male soldiers belonging to Gorkha (TB), Gorkha (IA), HAN (TB) and Indian lowlander (IA)

			Gorkha (TB)	Gorkha (IA)	HAN (TB)	Indian lowlander (IA)	Gorkha (TB) vs Gorkha (IA)	Gorkha (TB) vs HAN (TB)	Gorkha (TB) vs Indian lowlander (IA) (TB)	Gorkha (IA) vs HAN lowlander (IA)	Gorkha (IA) vs Indian lowlander (IA)	HAN (TB) vs Indian
SNP	Variants		Frequency						Level of significance (*p*)*			
*rs1799752*	*ACE I/D*	Genotype										
		*II*	0.427	0.459^a^	0.408^a^	0.254^a^						
		*ID*	0.467	0.417^a^	0.551^a^	0.504^a^	0.525	0.291	*0.000*	0.130	*0.000*	*0.003*
		*DD*	0.104	0.122^a^	0.040^a^	0.240^a^						
		Allele										
		*I*	0.671	0.673^a^	0.683^a^	0.506^a^	0.676	0.664	*0.000*	0.849	*1.0E-6*	*0.001*
		*D*	0.338	0.326^a^	0.316^a^	0.493^a^						
*rs1815739* *C/T*	*ACTN3* *Arg(R)577Ter(X)*	Genotype										
		*RR*	0.366	0.287^a^	0.189	0.180^a^	0.094	*0.010*	*0.000*	0.116	0.160	0.197
		*RX*	0.478	0.490^a^	0.648	0.555^a^						
		*XX*	0.155	0.222^a^	0.162	0.263^a^						
		Allele										
		*R*	0.605	0.520^a^	0.513	0.458^a^	*0.03*	*0.037*	*1.0E-6*	0.89	0.149	0.240
		*X*	0.394	0.479^a^	0.486	0.541^a^						
*rs8111989* *T/C*	*CKMM* *A/G NcoI*	Genotype										
		*AA*	0.655	0.475	0.594	0.455	*0.004*	0.608	*0.000*	0.289	0.245	0.065
		*AG*	0.296	0.445	0.351	0.388						
		*GG*	0.048	0.079	0.054	0.155						
		Allele										
		*A*	0.803	0.698	0.770	0.650	*0.001*	0.357	*1.0E-5*	0.133	0.317	*0.017*
		*G*	0.196	0.301	0.230	0.350						
*rs1799983* *G/T*	*eNOS* *Glu298Asp*	Genotype										
		*GG*	0.799	0.653	0.682	0.648	*0.000*	0.223	*0.000*	0.674	0.094	0.861
		*GT*	0.184	0.287	0.292	0.333						
		*TT*	0.016	0.588	0.024	0.018						
		Allele										
		*G*	0.891	0.797	0.829	0.814	*4.9E-5*	0.09	*0.0002*	0.51	0.554	0.754
		*T*	0.108	0.202	0.170	0.185						

*TB* Tibeto-Burman, *IA* Indo-Aryan, *HAN* high-altitude native from Ladakh

*Significance for genotypic and allelic frequency is assumed when *p* < 0.05. The significant *p* values are italicized

^a^Data from [10]

Higher frequency of *A* (major) allele of *CKMM A/G* and *G* (major) allele of *eNOS Glu298Arg*polymorphism was observed in the population (Table 5). Frequency of homozygous *AA* genotype of *CKMM A/G* was higher than homozygous mutant *GG* genotype in the population. Rais showed the highest *CKMM A* allele frequency compared to other Gorkha (Tibeto-Burman) groups. Frequency of homozygous *GG* genotype of *eNOS Glu298Arg* was also higher than homozygous mutant *TT* genotype. Limbus and Tamangs showed the highest *eNOS G* allele frequency compared to other groups. Homozygous mutant *TT* genotype of *eNOS Glu298Arg* was not observed in Tamang and Limbu groups (Table 5). Both the polymorphisms were in HWE. Of the 81 possible combinatorial genotypic profiles, 34 profiles were not observed. The predominant genotype combination was *ACE ID*/*ACTN3 RX*/*CKMM AA*/*eNOS GG* which was present in approximately 11.5% of the population (Additional file 2: Table S2). The optimal endurance-associated homozygous genotypic combination of *ACE II*/*ACTN3 XX*/*CKMM AA*/*eNOS GG* was present in nearly 4% of the male Gorkha soldier population (Additional file 2: Table S2). Although VO_2max_ values showed statistical difference within the subgroups, it did not show any statistically significant association with the genotypic profiles either individually or in combination (Additional file 3: Table S3 and Additional file 4: Table S4).

### Probability of optimal polygenic profile for endurance performance in the Gorkha (Tibeto-Burman) population

Typical genotypic frequencies were used for calculating the probability of possessing optimal genetic profile for endurance in the male Gorkha soldiers (Tibeto-Burman) as compared to lowlander Indian population (Table 7). The typical genotypic frequencies of the four polymorphisms ranged from 25% for *II* genotype of *ACE* to 65% of *GG* genotype of *eNOS* in lowlanders and 43% for *II* genotype of *ACE* to 80% of *GG* genotype of *eNOS* in male Gorkha soldiers (Table 7). Probability of any given lowlander soldier possessing the “favourable” genotype for endurance at one locus (*II* genotype of *ACE*) was ~25% which was reduced to ~6.5% when the second polymorphism (*XX* genotype of *ACTN3*) was added. The probability of any lowlander possessing the optimal polygenic profile for endurance at all four loci further reduced to 1.88% with an approximate odds ratio of 1:78 (i.e., one in 78 individuals) (Table 7). In the male Gorkha soldiers (Tibeto-Burman), probability of possessing the favourable genotype for endurance for one polymorphism (*II* genotype of *ACE*) was ~43% which was reduced to ~6.5% when the second polymorphism (*XX* genotype of *ACTN3*) was added. The probability of any male Gorkha soldier possessing the optimal polygenic profile for endurance at all four loci was ~3.35% with an approximate odds ratio of 1:30 (i.e., one in 30 individuals) (Table 7). The typical endurance-associated optimal genotypic frequency in the male Gorkha soldiers (Tibeto-Burman) ranged from 15% for *XX* genotype of *ACTN3* to 80% of *GG* genotype of *eNOS* (Table 8). Frequency distribution of the optimal endurance genotype in the male Gorkha soldiers and other Asian and Caucasian populations is shown in Table 8.

**Table 7:** Probability of possessing “optimal” genetic profile by number of polymorphism in the male Gorkha (TB) and Indian lowlander (IA) soldiers

Polymorphisms influencing endurance performance	New gene included at each stage	Gorkha (Tibeto-Burman)			Indian lowlander		
		Typical frequency (%) of “optimal” genotype	Probability of possessing ‘perfect’ profile		Typical frequency (%) of “optimal” genotype	Probability of possessing “perfect” profile	
			% chance	Approximate odds ratio		% chance	Approximate odds ratio
1.	*ACE*	43	43.00	1:2	25^a^	25.00	1:4
2.	*ACTN3*	15	6.45	1:15	26^a^	6.50	1.15
3.	*CKMM*	65	4.19	1:23	45	2.99	1:35
4.	*eNOS*	80	3.35	1:30	65	1.88	1:78

Data obtained from a data set of 100,000 hypothetical male Gorkha (TB) and Indian lowlander (IA), each with a randomly generated genetic profile for all four polymorphisms based on the typical frequency of each genotype

*TB* Tibeto-Burman, *IA* Indo-Aryan

^a^Genotypic frequencies taken from [10]

**Table 8:** Frequency distribution of “optimal” endurance genotype in male Gorkha soldiers and other male Indian soldiers

Gene	Symbol	Polymorphism	Genotype (2 “optimal” endurance genotype)	Frequency (%)							
				Gorkha (TB)	Gorkha (IA)	HAN (TB)	Indian lowlander (IA)	Gujrati Houston (GIH, IA)	Han Chinese Beijing (CHB, ST)	JPT	Caucasian (CEU)
Angiotensin converting enzyme (Peptidyl dipeptidase A)	*ACE*	*287 bp I/D (rs1799752)*	0 = *DD*, 1 = *ID*, 2 = *II*	10, 47, 43	12, 42, 46^a^	04, 55, 41^a^	24, 51, 25^a^	–	12, 45, 43^d^	12,47,41^f^	30,51,19^g^
Alpha actinin 3	*ACTN3*	*Arg(R)577Ter(X) (rs1815739)*	0 = *RR*, 1 = *RX*, 2 = *XX*	37, 48, 15	25, 52, 23^a^	19, 65, 16	18, 56, 26^a^	14, 59, 27^c^	43, 39, 17^c^	21, 61, 17^c^	20, 58, 22 ^c^
Creatine kinase, muscle	*CKMM*	*A/G NcoI (rs8111989)*	0 = *GG*, 1 = *AG*, 2 = *AA*	05, 30, 65	08, 45, 47	05, 35, 60	16, 39, 46	22, 39, 38^c^	04, 34, 61^c^	05, 27, 68^c^	05, 43, 51 ^c^
Endothelial nitric oxide synthase	*eNOS*	*Glu 298Asp (rs1799983)*	0 = *TT*, 1 = *GT*, 2 = *GG*	02, 18, 80	06, 29, 65	02, 26, 72^b^	02, 33, 65	–	0, 22, 78^e^	14, 86, 0^c^	08, 51, 40 ^c^

*TB* Tibeto-Burman, *IA* Indo-Aryan, *HAN* high-altitude natives from Ladakh, *ST* Sino-Tibetan, *CHB* Han Chinese, Beijing, *CEU* Utah residents with northern and western European ancestry, *GIH* Gujrati Indians in Houston, Texas, *JPT* Japanese in Tokyo, Japan

^a, b, d, f, g^Values from references [10, 23, 73–75]

^c^Data from HapMapPhase 3

^e^Data from the International HapMap Project

**Fig. 1:**
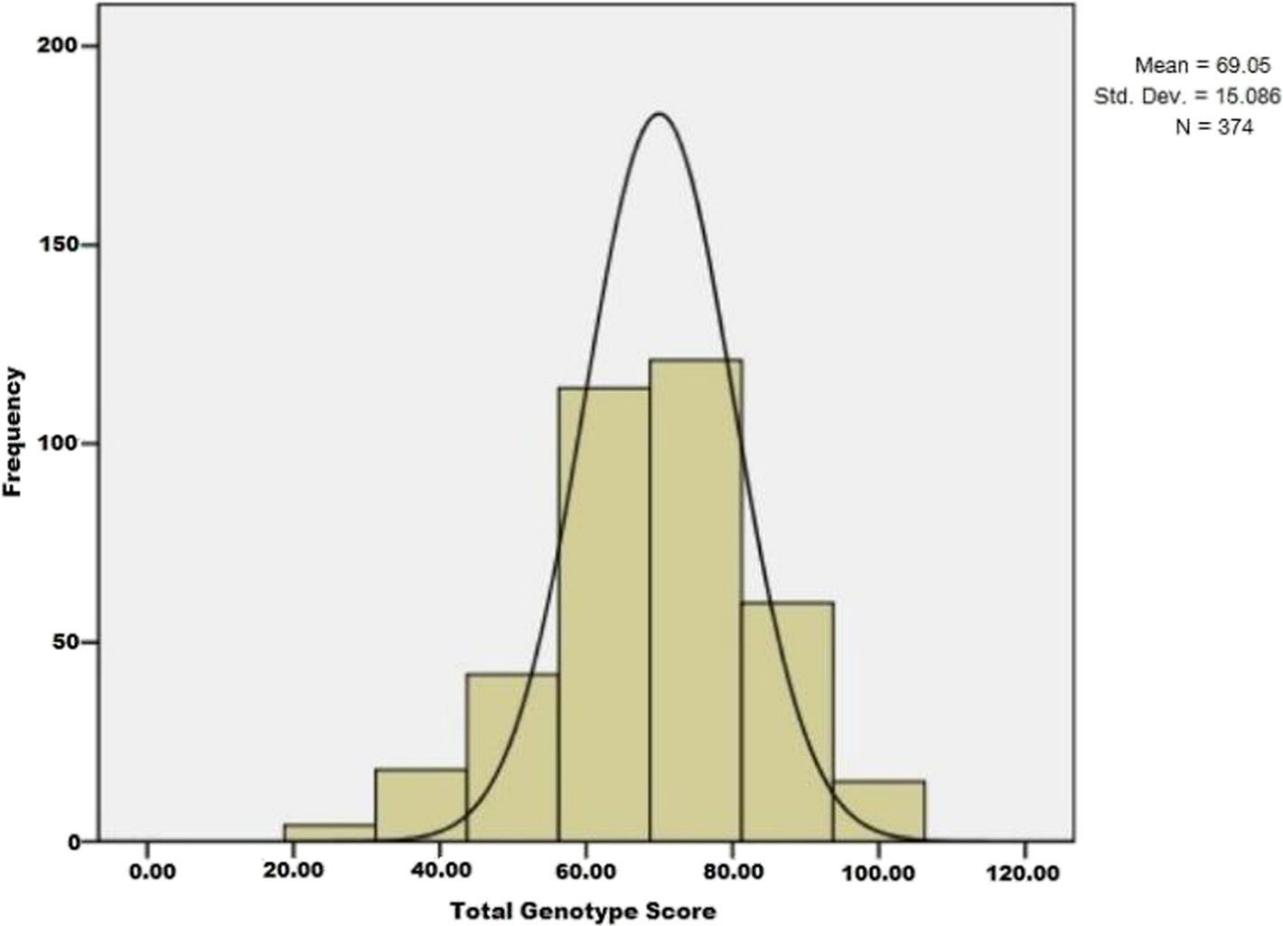
Frequency distribution of total genotypes score (TGS) in male Gorkha soldiers (*n* = 374)

The mean ± SD of total genotype score (TGS) was 69.05 ± 15.08 and kurtosis statistics 0.106 ± 0.252, and distribution was shifted towards the right in the male Gorkha soldiers (Fig. 1). About 4% of the male Gorkha soldiers (15 individuals out of 374) exhibited an optimal TGS (100) for endurance with another 16% of the population (60 individuals out of 374) showing a TGS of ~87 (Fig. 1, Additional file 5: Table S5). The lowest limit of genetic potential for endurance in male Gorkha soldiers was TGS ~25 with only 4 individuals having this score. On scrutiny, it was seen that two of these four individuals possessed *ACE DD*/*ACTN3 RR* genotypic combination, another possessed *ACE ID*/*ACTN3 RR* and the fourth one possessed *ACE DD*/*ACTN3RX* (Additional file 6: Table S6), genotypic combinations which are suggested to be favourably associated with muscle strength/power phenotypes [29]. Comparison of TGS (maximum 100 and minimum 25) with physiological characteristics (SBP, DBP and HR) and VO_2max_ did not show any statistical difference (Additional file 7: Table S7).

**Fig. 2:**
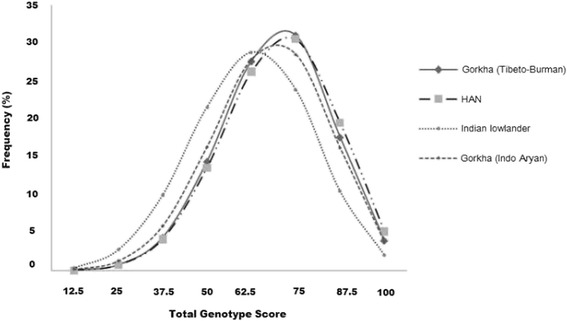
Frequency distribution of total genotype scores (TGS) derived from dataset of 100,000 randomly generated individuals of Indian lowlanders (IA), high-altitude native (TB), Gorkhas (IA) and Gorkhas (TB)

Frequency distribution of TGS calculated from the virtual samples is depicted in Fig. 2. In the simulated population of Gorkhas (Tibeto-Burman), mean ± SD TGS was 69.01 ± 15.40 and kurtosis statistics (−) 0.094 ± 0.015 (SE); 4% (4046 individuals out of 100,000) showed TGS 100 while 17.6% (17,591 individuals out of 100,000) had TGS 87 (Fig. 2) corroborating the observation obtained from the representative 374 participants of the present study (4% with TGS 100 and 16% with TGS of 87 (Fig. 1, Additional file 5: Table S5). In the simulated population of high-altitude natives, predicted mean ± SD TGS was 70.06 ± 15.66 and kurtosis statistics (−) 0.168 ± 0.015 (SE) with 5.2% (5174 individuals out of 100,000) having optimal TGS 100 and another 19.5% (19,487 individuals out of 100000) having TGS 87 (Fig. 2). In simulated Gorkha (Indo-Aryan) population, mean ± SD TGS was 67.44 ± 16.10 and kurtosis statistics (−) 0.176 ± 0.015 (SE); TGS 100 was observed in 3.8% (3761 out of 100,000 individuals), and TGS 87 was observed in 16.2% (16,199 individuals out of 100,000) (Fig. 2). In the simulated population of Indian lowlanders, the predicted mean ± SD TGS was 62.46 ± 16.66 and kurtosis statistics (−) 0.217 ± 0.015 (SE); TGS 100 was observed in 2.1% (2061 out of 100,000 individuals) and TGS 87 was in 10.5% (10530 individuals out of 100000) (Fig. 2). Number of Gorkha soldiers with TGS 100 was significantly higher than the Indian lowlander soldiers with TGS 100 (*p* < 0.0001) (2 × 2 contingency table; www.graphpad.com/quickcalcs/contingency2/).

## Discussion

The Gorkha population is largely an understudied population and genetic information on the population is scanty. This study reports for the first time the genotypic and allelic frequency distribution of endurance-related four polymorphic markers in the male Gorkha soldiers of Tibeto-Burman linguistic cluster and the polygenic endurance potential of the population. Ethnic heterogeneity with respect to *ACE I/D* polymorphism was noted with a trend of higher frequency of *ACE II* genotype and *I* allele in Tamangs. It may be mentioned here that the Gorkhas included in this study were main ethnics (tribes): Gurungs, Magars, Rais, Tamangs and Limbus. They look similar but are very different ethnics and could be having their own distinct genetic makeup. Thus, the frequency differences within the subgroups (although not significant after Bonferroni correction, *p* < 0.00250), could be attributed to their evolutionary adaptation. The observed genotypic frequency of *ACE I/D* in Tamangs was in agreement with frequency distribution reported from inhabitants of Kotyang, majority of whom were Tamangs [30]. Tamangs are indigenous inhabitants of the western Himalayan regions and one of the major Tibeto-Burman speaking communities who trace their ancestry to Tibet and further back to Mongolia (*Tamang people. World Public Library-eBooks*). Higher *I* allele frequency in Tamangs, similar to that found in Sherpas, suggests enhanced physical activity in the group. Predominance of homozygous *II* genotype and *I* allele of *ACE* in Gorkhas in the present study is in agreement with that observed in Gorkhas reported earlier, [10] majority of whom belonged to Indo-Aryan ethnicity. Predominance of *ACE I* allele in the Gorkhas suggests endurance and muscle efficiency [31, 32], the key determinants of performance and also associated with enhanced performance at high altitudes [31]. *ACE I* allele influences human physical performance and trainability [33, 34] and is also related to cardiorespiratory efficiency [35, 36]; although, contrary reports also exist showing no relationship between *ACE I/D* polymorphism and cardiorespiratory fitness [37].

*ACTN3* gene is associated with performance and genotype across multiple cohorts of elite power athletes and also supported by gene knockout mouse model [38]. *Ter* (*X*) allele affects endurance ability of elite athletes while *Arg* (*R*) allele affects sprinting [13, 39]. It would be interesting to further investigate the polygenic potential of *ACTN3 RR* genotype (associated with sprinting) along with power-related muscle performance genes in this population. ACTN3 *Ter* (*X*) allele and the *TerTer* (*XX*) genotypes are significantly associated with certain groups of elite endurance athletes [14, 40]. The frequency distribution of *Ter (X)* allele in the male Gorkha soldiers is observed to be 0.394 with genotype frequency being 0.155 *XX* and 0.366 *RR*. Interestingly, the *XX* genotype frequency in the male Gorkha soldiers was observed to be lower than Gorkha and Indian lowlander soldiers belonging to Indo-Aryan linguistic phylum as well as Caucasians (Table 8). Similar to the frequency observed in male Gorkha soldiers (Tibeto-Burman), the *XX* genotype frequency has been reported to be ~0.170 in other Asian populations viz., HAN Chinese (CHB, Sino-Tibetan linguistic phylum) [41] and Japanese (HapMap Phase 3) while in Caucasians, the *XX* frequency is 0.22 (HapMap Phase 3). At the moment, we do not have an explanation for less *XX* genotype frequency in the Gorkha (Tibeto-Burman) population compared to Caucasian population; this appears to be a population-specific difference between Caucasian and Asian (Tibeto-Burman/Sino-Tibetan) population. The derived 577X allele has been shown to increase in frequency with distance from Africa, reaching the highest frequencies on the American continent [42].

Creatine kinase is an important enzyme in energy metabolism which catalyzes phosphorylation of creatine to phosphocreatine, an energy storage molecule and source of ATP [43]. Muscle-specific creatine kinase gene (*CKMM*) correlates with athletic performance and *CKMM AA* genotype is considered as one of the genetic markers associated with predisposition to endurance-related sports activity [17]. The *CKMM A* allele probably influences gene expression and results in a decrease in muscle isoform of creatine kinase activity in myocytes leading to enhanced activation of oxidative phosphorylation and endurance development [17]. The frequency of the *CKMM A* allele varies from 85% in the Chinese population [44] to 68% in white Americans [16] with Caucasoids having 65–71% [45]. In the male Gorkha soldiers, frequency of *CKMM A* allele was 80.34% and *AA* genotype 65.50% which was comparable to that observed in high-altitude natives from India. Interestingly, frequency of *AA* genotype was significantly higher in Gorkhas of Tibeto-Burman ethnicity as compared to Gorkhas of Indo-Aryan ethnicity. Higher frequency distribution of *Glu(G)*allele of *eNOS* compared to that of *Asp(T)* allele in the Gorkhas, similar to the frequencies observed in high-altitude natives, suggests adaptive advantage of *Glu(G)* allele through increased production of nitric oxide (NO). Higher frequency of *Glu(G)* allele has been reported in Sherpas [46] and Quechuas of Andean *altpino* [47]. Higher exhaled NO has also been observed in Tibetan and Bolivian Aymara population [48, 49].

Maximum oxygen uptake capacity (VO_2max_), the maximal amount of oxygen per unit of time that can be delivered to the peripheral organs including the skeletal muscle (where it is used to sustain muscular contraction at peak exercise), is a bench mark measure of physical performance/work capacity [50]. It provides an index of functional reserve of the organ systems involved and limitation that can be encountered at peak exercise [51, 52]. In the present study, the overall VO_2max_ in the population was found to be 51.34 ± 7.22 ml kg ^−1^ min ^−1^ with Tamangs showing the highest maximal oxygen uptake compared to other subgroups (*p* < 0.05) (Table 4). Higher VO_2max_ is reflected in better performance in running, hill climbing and endurance work [53, 54]. VO_2max_ in the male Gorkha soldiers was higher as compared to the values reported from Indian general population [55–57] suggesting overall better endurance potential of Gorkhas (Tibeto-Burman) at the physiological level. We did not, however, observe statistically significant association between VO_2max_, and the studied polymorphisms in the Gorkhas either individually with four genetic variants or when combined genotype profile of each individual was analysed in the 122 volunteers who participated in the assessment of VO_2max_ (Additional file 3: Table S3 and Additional file 4: Table S4). It is probable that the small cohort of individuals for VO_2max_ may be limiting the interpretation of the result or it may also be probable that VO_2max_ is indeed not associated with the studied polymorphisms. Rankinen and co-workers [58] analysed elite endurance athletes with VO_2max_ values over 83 ml kg^−1^ and found no trend for excess *I* allele or low number of *DD* homozygotes of *ACE*. A genomic scan for maximal oxygen uptake in Caucasian families of the HERITAGE Family study reported many potential candidate gene loci on many chromosomal regions, but no linkage was observed on chromosome 17 on the *ACE* locus [59]. It appears logical to suggest that the study of influence of genotypes on the VO_2max_ should be conducted in an increased cohort of healthy individuals for genetic association to be more evident. In addition to maximal rate of oxygen uptake, at least two other endurance phenotypes (economy of movement and lactate/ventilator threshold) also contribute to endurance performance phenotype (time taken to travel a given distance) as seen in elite competition [11]. A fourth phenotype, namely oxygen uptake kinetics, may also be discretely related. These phenotypes are yet to be associated with specific genetic polymorphisms in a healthy adult population. Unbiased genome-wide approaches have been used in search for genomic region, transcripts and DNA variants linked or associated with endurance performance related trait [60–63]. Bouchard et al. [60] studied association of 324,611 SNPs with the response of VO_2max_ to endurance training in 473 Whites from HERITAGE. None of the SNPs reached genome-wide significance even though there were several SNPs moderately associated with VO_2max_ trainability [60]. A meta-analysis of genome-wide association study of two cohorts of elite endurance athletes and controls revealed only one statistically significant marker (*rs558129* at *GALNTL6* locus, *p* = 0.0002), and no panel of genomic variation common to the elite candidate athletic group was identified [64].

Human physical capability is influenced by many environmental and genetic factors, and it is generally accepted that physical performance phenotypes are highly polygenic [65, 66]. Potential for elite human physical performance is limited by the similarity of polygenic profiles with 99% of people differing by no more than seven genotypes from the typical profile [11]. The predicted mean TGS for favourable endurance profile in endurance elite athletes was significantly higher (70.2 ± 15.6) compared to general Spanish population (60.70 ± 12.21) and about 3000 individuals out of a total population of ~41 million people were predicted to have theoretically optimal polygenic profile (TGS = 100) for seven candidate genes [27]. In the simulated population of Gorkhas in the present study, mean TGS obtained was 69.01 ± 15.40 for favourable endurance. It was interesting to note that 4% of the population (15 individuals out of 374) exhibited an optimal TGS (100) for endurance with another 16% of the population (60 individuals out of 374) showing a TGS of ~87. Compared to Gorkhas, only 2 and 10% of Indian lowlanders had TGS 100 and TGS 87, respectively. Analysis of TGS 100 and TGS 87.5 between Gorkha (Tibeto-Burman) and Indian lowlanders, using 3 × 2 contingency table (www.vassarstats.net/newcs.html), demonstrated significant difference between the cohorts (*p* < 0.0001) suggesting an overall more favourable polygenic endurance profile in Gorkha population. With 4% of the male Gorkha soldiers (who are otherwise drawn from the general population) having the optimal endurance profile, this would mean that nearly 1.7 million, out of an estimated 43 million (31 million of Nepalese from Nepal and about 12 million Nepalese domiciled in India, Nepal Census 2011) would have the optimal endurance profile. Even if new candidate polymorphisms are scored, probability of presence of individuals with optimal genotypes for endurance still remains large in this population. The Gorkhas are thus genetically endowed with the endurance phenotypes; they have the genetic advantage with higher co-occurrence of *ACE II/ACTN3 XX/CKMM AA/eNOS GG* in the population. Genetic endowment coupled with high-intensity training which considerably increases maximal oxygen uptake, of young adolescent Gorkhas will definitely bring performance improvement in many sporting events. It may not be farfetched to postulate that such individuals can rewrite the world records. It is accepted that in addition to genetic potential and physical environment, a performance record is a function of economic and social opportunity. Individuals from small geographical area hold many world athletic records. Although genetic explanations is lacking, there are considerable regional and ethnic variations in typical frequencies of many genotypes e.g., frequency of *ACE II* is higher in some regions of Oceania than most of Europe while athletes of north and east African descent excel in endurance events and those of west African descent excel in sprint events [67]. Interestingly, 5% of high-altitude natives (Tibeto-Burman) also had TGS 100 and 19% had TGS 87 similar to the TGS frequency observed in Gorkhas (Tibeto-Burman). The observation presents a scope for us to state that Tibeto-Burman mountain population per se has higher TGS score linked to endurance genotype than the lowlander Indian population.

It may be argued that the soldiers used in the present study may not be representative of the entire Gorkha population as recruitment in Indian Army may be selective. For examining this issue, we compared the genotypic frequencies obtained from participants of Indian Army with those reported from general Indian population (Table 9). The genotypic frequency distribution in both the army and general population was not statistically different (except for *ACTN3*), substantiating the fact that the genotypic frequency distribution obtained from soldier population of the Indian Army is generally reflective of the frequency in general Indian population. As an extension of this observation, we state that the data obtained in the Gorkhas (Tibeto-Burman) in the present study reflects the general Gorkha population frequency.

**Table 9:** Comparison of genotypic and allelic frequency distribution between participants of the Indian Army and general Indian population

			Indo-Aryan			Dravidian			General Population		
			Indian Army	General population		Indian Army	General population		Indian Army	General population	
SNP	Variants	Genotype			*p* ^*^			*p* ^*^			*p* ^*^
*rs1799752*	*ACE* *I/D*	*II*	0.25^a^	0.26^b^	0.71	0.29^a^	0.37^e^	0.18			
		*ID*	0.50^a^	0.54^b^		0.53^a^	0.42^e^				
		*DD*	0.24^a^	0.20^b^		0.18^a^	0.21^e^				
		*Allele*									
		*I*	0.51^a^	0.53^b^	0.55	0.56^a^	0.58^e^	0.64			
		*D*	0.49^a^	0.46^b^		0.44^a^﻿	0.42^e^				
*rs1815739*	*ACTN3* *Arg(R)* *577Ter(X)*	*RR*	0.18^a^	0.16^c^	*0.04*	0.24^a^					
		*RX*	0.56^a^	0.45^c^		0.48^a^	NA	NA			
		*XX*	0.26^a^	0.39^c^		0.28^a^					
		*Allele*									
		*R*	0.46^a^	0.39^c^	*0.05*	0.48^a^					
		*X*	0.54^a^	0.61^c^		0.52^a^					
*rs1799983*	*eNOS* *Glu298Asp*	*GG*	0.64^i^	0.71^d^	0.12	0.73^i^	0.74^f^	0.45			
		*GT*	0.33^i^	0.27^d^		0.25^i^	0.26^f^				
		*TT*	0.01^i^	0.07^d^		0.01^i^	0.00^f^				
		*Allele*									
		*G*	0.81^i^	0.85^d^	0.90	0.86^i^	0.87^f^	0.76			
		*T*	0.18^i^	0.15^d^		0.14^i^	0.13^f^				
*rs1042713*	*ADRB2* *Arg16Gly*	*GG*							0.31^g^	0.25^k^	0.49
		*AG*							0.53^g^	0.53^k^	
		*AA*							0.16^g^	0.22^k^	
		*Allele*									
		*G*							0.56^g^	0.52^k^	0.25
		*A*							0.43^g^	0.48^k^	
*rs41317140*	*REN C/T*	*CC*	0.62^j^	0.69^h^	0.35						
		*CT*	0.35^j^	0.31^h^							
		*TT*	0.02^j^	0.00^h^							
		*Allele*									
		*C*	0.80^j^	0.84^h^	0.29						
		*T*	0.20^j^	0.16^h^							

Data for CKMM is not available in Indian general population

*NA* data not available

*Significance for genotypic and allelic frequency is assumed when *p* < 0.05. The significant *p* values are italicized

^a^[10]; ^b^[76]; ^c^[77]; ^d^[78]; ^e^[79]; ^f^[80]; ^g^[23]; ^h^[81]; ^i^Present study; ^j^unpublished; ^k^values computed by clubbing the data from [82] and [83]

### Limitations

The present study, however, is not without limitation as only four endurance-associated polymorphisms were studied. These four polymorphisms were chosen because of definitive information about their association with endurance, exercise performance, muscle performance, generation of rapid forceful contraction, high values of VO_2max_ and endurance elite status and also for reason that frequency data for these polymorphisms were available in the Indian population for comparison and computation of TGS. Further studies are required in larger cohorts and with additional endurance-related polymorphisms to evaluate the role of genetic variations in determining sports performance. It is also mentioned that in absence of objective data, we have used the extension of the observation seen in general Indian population and Indian army to state that the data obtained in the male Gorkha soldiers in the present study reflects the general Gorkha population frequency.

### Implications

The findings of the present study imply that young talented adolescents from the high hill population, having polygenic endurance profiles linked with higher TGS could be selected and given appropriate training in endurance-related sports activities with prospects of competing in Olympics and other sports events held in high-altitude environment. Earlier studies have shown that whole body physiological variables related to O_2_ consumption are influenced by the level of training of the individual [68–71]. It has been shown that long-term endurance training in a coordinated fashion impacts muscle epigenetics and affects thousands of methylation sites and genes associated with improvement in muscle function and health [72]. Appropriate training combined with favourable genetic profile will definitely be advantageous for achievement of elite athletic status by this sub-mountainous population.

## Conclusions

The present study provides a novel perspective on endurance genetics based on polygenic profiling in the Gorkha population. The study reports for the first time the genotypic and allelic frequencies of four endurance-associated genetic markers in the male Gorkha soldiers (Tibeto-Burman). The study also highlights that nearly 4% of the Gorkha soldiers (Tibeto-Burman) exhibit an optimal total genotypic score (100) for endurance, indicating genetic potential of this population for achieving excellence in endurance-related elite sports activities.

## Additional files

Additional file 1: Table S1. One-way ANOVA (PostHocTukey test) of VO_2max_ in five subpopulation of male Gorkha soldiers (TB). (DOCX 22 kb)

Additional file 2: Table S2. Frequency of combined genotype distribution in male Gorkha soldiers. (DOC 106 kb)

Additional file 3: Table S3. Descriptive statistics and comparative analysis of maximal oxygen uptake (VO_2 max_ in ml kg^−1^ min^−1^) between genotypes and groups. (DOC 36 kb)

Additional file 4: Table S4. Association analysis of combinatorial genotype profiles with maximal oxygen uptake (VO_2max_ in ml kg^−1^ min^−1^). (DOC 36 kb)

Additional file 5: Table S5. Frequency distribution of total genotype score (TGS) from male Gorkha soldiers (*n* = 374). (a) Tabular information of TGS. (b) Graphical representation of TGS. (DOC 85 kb)

Additional file 6: Table S6. Total genotype score and percentage of frequency for all the possible combinations of four polymorphisms in male Gorkha soldiers. (DOC 89 kb)

Additional file 7: Table S7. Comparison of physiological characteristics and VO_2max_ in male Gorkha soldiers grouped according to total genotype score (TGS). (DOCX 32 kb)

## Footnotes

1 The Gorkhas derive their name from Guru Gorakhanath of eighth century. The shrine dedicated to Guru Gorakhnath was part of Gorkha Durbar built on a rocky crag in Nepal in 1639 from where the Gorkhas began their soldiering and where the ancient army called “Gurujiko Paltan” was raised. The 400-year-old shrine dedicated to Guru Gorkhnath was devastated in the massive earthquake that struck Nepal on 25 April 2015.
