# Supplementary material for: Polygenic study of endurance-associated genetic markers ACE I/D, ACTN3 Arg(R)577Ter(X), CKMM A/G NcoI and eNOS Glu(G)298Asp(T) in male Gorkha soldiers
Source: Sports Med Open. 2017 Apr 26;3:17. doi: 10.1186/s40798-017-0085-0 (PMC5405041; doi:10.1186/s40798-017-0085-0)
Supplement: Supplementary file 1 — One-way ANOVA (PostHocTukey test) of VO2max in five subpopulation of male Gorkha soldiers (TB). (DOCX 22 kb) [file 40798_2017_85_MOESM1_ESM.docx]

**Polygenic study of endurance associated genetic markers *ACE I/D, ACTN3 Arg(R)577Ter(X)*, *CKMM A/G NcoI* and *eNOS Glu(G)298Asp(T)* in male Gorkha soldiers**

Journal Name: Sports Medicine

SeemaMalhotra, KiranPreet, ArvindTomar*, ShwetaRawat, Sayar Singh, Inderjeet Singh, L. Robert Varte, TirthankarChatterjee, M.S Pal and Soma Sarkar†

Defence Institute of Physiology and Allied Sciences (DIPAS), Ministry of Defence. Government of India, Lucknow Road, Delhi 110054.*Defence Research and Development Establishment (DRDE).Ministry of Defence, Government of India, Jhansi Road, Gwalior 474002, Madhya Pradesh.

†**CORRESPONDING AUTHOR:**

email: [soma_sarkar2000@yahoo.com](mailto:soma_sarkar2000@yahoo.com)

Table S1**:** One way ANOVA (PostHocTukey test) of VO_2max_ in five subpopulation of male Gorkha soldiers (TB)

| **ANOVA** | | | | | |
| --- | --- | --- | --- | --- | --- |
| VO_2max_ | | | | | |
|  | Sum of Squares | df | Mean Square | F | Sig. |
| Between Groups | 1782.967 | 4 | 445.742 | 8.956 | .000 |
| Within Groups | 5823.293 | 117 | 49.772 |  |  |
| Total | 7606.261 | 121 |  |  |  |

| **Post Hoc Tests (Tukey HSD)** | | | | | | |
| --- | --- | --- | --- | --- | --- | --- |
| VO_2max_ | | | | | | |
| (I) group | (J) group | Mean Difference (I-J) | Std. Error | Sig. | 95% Confidence Interval | |
|  |  |  |  |  | Lower Bound | Upper Bound |
| 1.00 | 2.00 | -1.40959 | 1.77789 | .932 | -6.3357 | 3.5166 |
|  | 3.00 | -6.30243^*^ | 1.94153 | .**013** | -11.6820 | -.9229 |
|  | 4.00 | -9.51011^*^ | 2.09061 | **.000** | -15.3027 | -3.7175 |
|  | 5.00 | -8.78344^*^ | 2.09061 | .**000** | -14.5761 | -2.9908 |
| 2.00 | 1.00 | 1.40959 | 1.77789 | .932 | -3.5166 | 6.3357 |
|  | 3.00 | -4.89284 | 1.92856 | .089 | -10.2365 | .4508 |
|  | 4.00 | -8.10052^*^ | 2.07857 | .**002** | -13.8598 | -2.3413 |
|  | 5.00 | -7.37385^*^ | 2.07857 | **.005** | -13.1331 | -1.6146 |
| 3.00 | 1.00 | 6.30243^*^ | 1.94153 | **.013** | .9229 | 11.6820 |
|  | 2.00 | 4.89284 | 1.92856 | .089 | -.4508 | 10.2365 |
|  | 4.00 | -3.20768 | 2.22015 | .600 | -9.3592 | 2.9439 |
|  | 5.00 | -2.48101 | 2.22015 | .797 | -8.6326 | 3.6705 |
| 4.00 | 1.00 | 9.51011^*^ | 2.09061 | **.000** | 3.7175 | 15.3027 |
|  | 2.00 | 8.10052^*^ | 2.07857 | **.002** | 2.3413 | 13.8598 |
|  | 3.00 | 3.20768 | 2.22015 | .600 | -2.9439 | 9.3592 |
|  | 5.00 | .72667 | 2.35164 | .998 | -5.7892 | 7.2425 |
| 5.00 | 1.00 | 8.78344^*^ | 2.09061 | **.000** | 2.9908 | 14.5761 |
|  | 2.00 | 7.37385^*^ | 2.07857 | **.005** | 1.6146 | 13.1331 |
|  | 3.00 | 2.48101 | 2.22015 | .797 | -3.6705 | 8.6326 |
|  | 4.00 | -.72667 | 2.35164 | .998 | -7.2425 | 5.7892 |
| *The mean difference is significant at the 0.05 level. TB: Tibeto-Burman. | | | | | | |

Subpopulation Group

Gurung 1

Magar 2

Rai 3

Tamang 4

Limbu 5
