# Supplementary material for: Polygenic study of endurance-associated genetic markers ACE I/D, ACTN3 Arg(R)577Ter(X), CKMM A/G NcoI and eNOS Glu(G)298Asp(T) in male Gorkha soldiers
Source: Sports Med Open. 2017 Apr 26;3:17. doi: 10.1186/s40798-017-0085-0 (PMC5405041; doi:10.1186/s40798-017-0085-0)

**Polygenic study of endurance associated genetic markers *ACE I/D, ACTN3 Arg(R)577Ter(X)*, *CKMM A/G NcoI* and *eNOS Glu(G)298Asp(T)* in male Gorkha soldiers**

Journal Name: Sports Medicine

Seema Malhotra, Kiran Preet, Arvind Tomar*, Shweta Rawat, Sayar Singh, Inderjeet Singh, L. Robert Varte, Tirthankar Chatterjee, M.S Pal and Soma Sarkar†

Defence Institute of Physiology and Allied Sciences (DIPAS), Ministry of Defence. Government of India, Lucknow Road, Delhi 110054. *Defence Research and Development Establishment (DRDE). Ministry of Defence, Government of India, Jhansi Road, Gwalior 474002, Madhya Pradesh.

†**CORRESPONDING AUTHOR:**

email: [soma_sarkar2000@yahoo.com](mailto:soma_sarkar2000@yahoo.com)


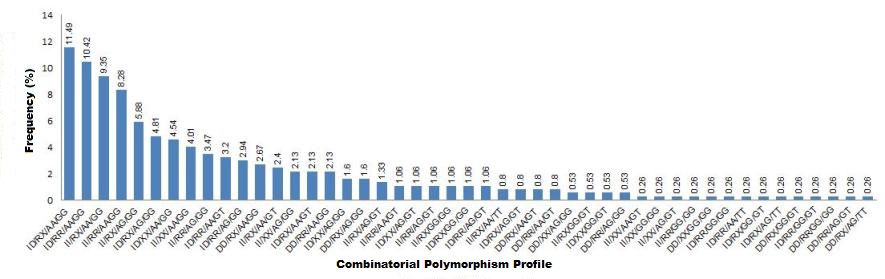
Table S2: Frequency of combined genotype distribution in male Gorkha soldiers


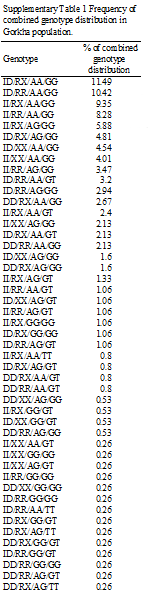

Supplement: Supplementary file 2 — Frequency of combined genotype distribution in male Gorkha soldiers. (DOC 106 kb) [file 40798_2017_85_MOESM2_ESM.doc]
