# Supplementary material for: Polygenic study of endurance-associated genetic markers ACE I/D, ACTN3 Arg(R)577Ter(X), CKMM A/G NcoI and eNOS Glu(G)298Asp(T) in male Gorkha soldiers
Source: Sports Med Open. 2017 Apr 26;3:17. doi: 10.1186/s40798-017-0085-0 (PMC5405041; doi:10.1186/s40798-017-0085-0)
Supplement: Supplementary file 3 — Descriptive statistics and comparative analysis of maximal oxygen uptake (VO2 max in ml kg−1 min−1) between genotypes and groups. (DOC 36 kb) [file 40798_2017_85_MOESM3_ESM.doc]

**Polygenic study of endurance associated genetic markers *ACE I/D, ACTN3 Arg(R)577Ter(X)*, *CKMM A/G NcoI* and**

***eNOS Glu(G)298Asp(T)* in male Gorkha soldiers**

Journal Name: Sports Medicine

Seema Malhotra, Kiran Preet, Arvind Tomar*, Shweta Rawat, Sayar Singh, Inderjeet Singh, L. Robert Varte, Tirthankar Chatterjee, M.S Pal and Soma Sarkar†

Defence Institute of Physiology and Allied Sciences (DIPAS), Ministry of Defence. Government of India, Lucknow Road, Delhi 110054. *Defence Research and

Development Establishment (DRDE). Ministry of Defence, Government of India, Jhansi Road, Gwalior 474002, Madhya Pradesh.

†**CORRESPONDING AUTHOR:**

email: [soma_sarkar2000@yahoo.com](mailto:soma_sarkar2000@yahoo.com)

Table S3 Descriptive statistics and comparative analysis of maximal oxygen uptake (VO_2 max_ in ml. kg^-1^. min^-1^) between

genotypes and groups.

_________________________________________________________________________________________________________

Gurung Magar Rai Tamang Limbu VO_2 max_ Effects Interaction

(n=31) (n=32) (n=23) (n=18) (n=18) Groups Genotype

_________________________________________________________________________________________________________

*ACE I/D Overall* 46.56±8.18 47.97±6.08 52.86±6.19 56.07±8.27 55.34±6.21

*II* 48.27±8.08 48.36±7.33 53.32±6.75 58.68±10.95 56.80±6.85 0.000 0.163 0.986
 *ID* 44.39±8.40 47.16±6.10 51.68±5.69 54.77±6.77 54.42±5.91

*DD* 46.07±8.64 50.42±3.12 55.89±0 00 00

*ACTN3 Overall* 46.56±8.18 47.97±6.08 52.86±6.19 56.07±8.27 55.34±6.21

*RR* 46.95±7.82 51.77±5.14 52.74±4.84 54.95±11.47 55.88±7.34 0.000 0.744 0.807

*RX* 46.19±9.42 44.97±6.63 53.23±8.67 57.01±4.06 55.18±5.78

*XX* 46.18±6.46 49.37±4.08 52.52±7.32 57.57±1.68 52.53±0

*CKMM Overall* 46.56±8.18 47.97±6.08 52.86±6.19 56.07±8.27 55.34±6.21

*AA* 46.06±7.90 47.15±6.52 52.81±6.35 57.22±9.51 54.88±5.88 0.000 0.540 0.784

*AG* 47.46±11.71 50.33±3.94 57.57±0 54.63±6.76 55.92±6.95

*GG* 47.82±4.44 44.15±8.20 49.17±0 00 00

*eNOS Overall* 46.56±8.18 47.97±6.08 52.86±6.19 56.07±8.27 55.34±6.211

*GG* 47.01±8.78 47.72±6.08 51.92±5.86 54.95±11.47 53.71±6.11 0.000 0.683 0.189

*GT* 44.70±5.19 48.55±5.58 57.32±6.51 57.01±4.06 60.84±2.18

*TT* 00 49.00±00 00 57.57±1.68 00

_________________________________________________________________________________________________________

Note: Analysis of variance did not show statistical significance difference in VO_2 max_ with genotypes
