# Supplementary material for: Polygenic study of endurance-associated genetic markers ACE I/D, ACTN3 Arg(R)577Ter(X), CKMM A/G NcoI and eNOS Glu(G)298Asp(T) in male Gorkha soldiers
Source: Sports Med Open. 2017 Apr 26;3:17. doi: 10.1186/s40798-017-0085-0 (PMC5405041; doi:10.1186/s40798-017-0085-0)
Supplement: Supplementary file 4 — Association analysis of combinatorial genotype profiles with maximal oxygen uptake (VO2max in ml kg−1 min−1). (DOC 36 kb) [file 40798_2017_85_MOESM4_ESM.doc]

**Polygenic study of endurance associated genetic markers *ACE I/D, ACTN3 Arg(R)577Ter(X)*, *CKMM A/G NcoI* and *eNOS Glu(G)298Asp(T)* in male Gorkha soldiers**

Journal Name: Sports Medicine

Seema Malhotra, Kiran Preet, Arvind Tomar*, Shweta Rawat, Sayar Singh, Inderjeet Singh, L. Robert Varte, Tirthankar Chatterjee, M.S Pal and Soma Sarkar†

Defence Institute of Physiology and Allied Sciences (DIPAS), Ministry of Defence. Government of India, Lucknow Road, Delhi 110054. *Defence Research and Development Establishment (DRDE). Ministry of Defence, Government of India, Jhansi Road, Gwalior 474002, Madhya Pradesh.

†**CORRESPONDING AUTHOR:**

email: [soma_sarkar2000@yahoo.com](mailto:soma_sarkar2000@yahoo.com)

Table S4 Association analysis of combinatorial genotype profiles

with maximal oxygen uptake (VO_2max_ in ml. kg^-1^. min^-1^)

___________________________________________________________________

Genotype Percent VO_2max_ p

Combination population (mean± SD)

___________________________________________________________________

II /RX/AA/GT 3(2.45) 62.07±3.48

II/ XX/GA/GT 1(0.81) 57.57±0

II/ RX/ GA/GG 7(5.73) 55.98±6

ID/XX/GG/GT 1(0.81) 55.89±0

DD/ RR/AA/GG 2(1.63) 55.05±1.18

II/RR/AA/GG 11(9.01) 54.4±8.26

ID/RR/GA/GT 1(0.81) 54.4±0

II/RX/GA/GT 2(1.63) 53.74±14.33

ID/RX/GA/GG 4(3.27) 53.29±0.69

ID/RR/AA/GG 14(11.47) 52.77±8.34

ID/XX/GA/GG 4(3.27) 51.56±7.80

II/RR/AA/ GT 2(1.63) 51.19±9.98

ID/RX/GG/GG 2(1.63) 51.15±1.62 0.641

II/RR/GA/GG 7(5.73) 51.03±9.62

II/XX/AA/GG 5(4.09) 50.43±7.36

II/RX/GG/GT 1(0.81) 50±0

ID/RR/GG/GT 1(0.81) 50±0

ID/XX/GA/G T 2(1.63) 49.65±3.74

ID/RR/AA/GT 6(4.91) 49.28±5.39

ID/XX/AA/GG 5(4.09) 49.27±4.44

II/RR/GG/GG 1(0.81) 49.17±0

ID/RR/GA/GG 4(3.27) 49.13±12.38

II/RX/AA/ TT 1(0.81) 49±0

II/XX/GA/GG 1(0.81) 49±0

DD/XX/GA/GG 2(1.63) 49±2.82

DD/RR/AA /GG 2(1.63) 48.85±1.62

ID/RX/AA/GT 3(2.45) 48.25±9.53

II/RX/AA/GG 7(5.73) 47.08±8.12

DD/RR/GG/GG 1(0.81) 47±0

DD/RR/AA/GT 2(1.63) 46.7±13.71

ID/RX/AA/GG 14(11.47) 46.49±9.42

ID/RX/GG/GT 1(0.81) 44±0

II/RX/GG/GG 2(1.63) 37.3±6.64

___________________________________________________________________

Genotype combinations are written in the alphabetical order: ACE/ACTN3/CKMM/eNOS.

p value calculated by univariant analysis of variance (ANOVA) using SPSS Statistics

version 17.0.
