# Supplementary material for: Polygenic study of endurance-associated genetic markers ACE I/D, ACTN3 Arg(R)577Ter(X), CKMM A/G NcoI and eNOS Glu(G)298Asp(T) in male Gorkha soldiers
Source: Sports Med Open. 2017 Apr 26;3:17. doi: 10.1186/s40798-017-0085-0 (PMC5405041; doi:10.1186/s40798-017-0085-0)
Supplement: Supplementary file 6 — Total genotype score and percentage of frequency for all the possible combinations of four polymorphisms in male Gorkha soldiers. (DOC 89 kb) [file 40798_2017_85_MOESM6_ESM.doc]

| **Polygenic study of endurance associated genetic markers *ACE I/D, ACTN3 Arg(R)577Ter(X)*, *CKMM A/G NcoI* and *eNOS Glu(G)298Asp(T)* in male Gorkha soldiers**  Journal Name: Sports Medicine  Seema Malhotra, Kiran Preet, Arvind Tomar*, Shweta Rawat, Sayar Singh, Inderjeet Singh, L. Robert Varte, Tirthankar Chatterjee, M.S Pal and Soma Sarkar†  Defence Institute of Physiology and Allied Sciences (DIPAS), Ministry of Defence. Government of India, Lucknow Road, Delhi 110054. *Defence Research and Development Establishment (DRDE). Ministry of Defence, Government of India, Jhansi Road, Gwalior 474002, Madhya Pradesh.  †**CORRESPONDING AUTHOR:**  email: [soma_sarkar2000@yahoo.com](mailto:soma_sarkar2000@yahoo.com)  Table S6 Total genotype score and percentage of frequency for all the possible combinations of four polymorphisms in male Gorkha soldiers | | | | |
| --- | --- | --- | --- | --- |
| S.No. | Genotype | Total Genotype Score | No. of individual n=374 | Frequency % |
| 1 | II/XX/AA/GG | 100 | 15 | 4.01 |
| 2 | II/RX/AA/GG | 87.5 | 35 | 9.35 |
| 3 | ID/XX/ AA/GG | 87.5 | 17 | 4.54 |
| 4 | II/XX/GG/AG | 87.5 | 8 | 2.13 |
| 5 | II/XX/GT/AA | 87.5 | 1 | 0.26 |
| 6 | ID/RX/AA/GG | 75 | 43 | 11.49 |
| 7 | II/RR/AA/GG | 75 | 31 | 8.28 |
| 8 | II/RX/AG/GG | 75 | 22 | 5.88 |
| 9 | II/RX/GT/AA | 75 | 9 | 2.4 |
| 10 | ID/XX/GG/AG | 75 | 6 | 1.6 |
| 11 | II/XX/GG/GG | 75 | 1 | 0.26 |
| 12 | II/XX/GT/AG | 75 | 1 | 0.26 |
| 13 | II/XX/TT/AA | 75 | 0 | 0 |
| 14 | DD/XX/ AA/GG | 75 | 0 | 0 |
| 15 | ID/RR/AA/GG | 62.5 | 39 | 10.42 |
| 16 | ID/RX/GG/AG | 62.5 | 18 | 4.81 |
| 17 | II/RR/GG/AG | 62.5 | 13 | 3.47 |
| 18 | DD/RX/ AA/GG | 62.5 | 10 | 2.67 |
| 19 | ID/RX/GT/AA | 62.5 | 8 | 2.13 |
| 20 | II/RX/GT/AG | 62.5 | 5 | 1.33 |
| 21 | II/RR/GT/AA | 62.5 | 4 | 1.06 |
| 22 | ID/XX/GT/AG | 62.5 | 4 | 1.06 |
| 23 | II/RX/TT/AA | 62.5 | 3 | 0.8 |
| 24 | DD/XX/GG/AG | 62.5 | 2 | 0.53 |
| 25 | II/XX/GT/GG | 62.5 | 0 | 0 |
| 26 | II/XX/TT/AG | 62.5 | 0 | 0 |
| 27 | ID/XX/GG/GG | 62.5 | 0 | 0 |
| 28 | ID/XX/TT/AA | 62.5 | 0 | 0 |
| 29 | DD/XX/GT/AA | 62.5 | 0 | 0 |
| 30 | ID/RR/GT/AA | 50 | 12 | 3.2 |
| 31 | ID/RR/GG/AG | 50 | 11 | 2.94 |
| 32 | DD/RR/ AA/GG | 50 | 8 | 2.13 |
| 33 | DD/RX/GG/AG | 50 | 6 | 1.6 |
| 34 | II/RR/GT/AG | 50 | 4 | 1.06 |
| 35 | II/RX/GG/GG | 50 | 4 | 1.06 |
| 36 | ID/RX/GG/GG | 50 | 4 | 1.06 |
| 37 | ID/RX/GT/AG | 50 | 3 | 0.8 |
| 38 | DD/RX/GT/AA | 50 | 3 | 0.8 |
| 39 | II/RX/GT/GG | 50 | 2 | 0.53 |
| 40 | ID/XX/GT/GG | 50 | 2 | 0.53 |
| 41 | II/RR/GG/GG | 50 | 1 | 0.26 |
| 42 | DD/XX/GG/GG | 50 | 1 | 0.26 |
| 43 | II/RR/TT/AA | 50 | 0 | 0 |
| 44 | II/RX/TT/AG | 50 | 0 | 0 |
| 45 | II/XX/TT/GG | 50 | 0 | 0 |
| 46 | ID/RX/TT/AA | 50 | 0 | 0 |
| 47 | ID/XX/GT/GG | 50 | 0 | 0 |
| 48 | ID/XX/TT/AG | 50 | 0 | 0 |
| 49 | DD/XX/GT/AG | 50 | 0 | 0 |
| 50 | DD/XX/TT/AA | 50 | 0 | 0 |
| 51 | ID/RR/GT/AG | 37.5 | 4 | 1.06 |
| 52 | DD/RR/GT/AA | 37.5 | 3 | 0.8 |
| 53 | DD/RR/GG/AG | 37.5 | 2 | 0.53 |
| 54 | ID/RR/GG/GG | 37.5 | 1 | 0.26 |
| 55 | ID/RR/TT/AA | 37.5 | 1 | 0.26 |
| 56 | ID/RX/GT/GG | 37.5 | 1 | 0.26 |
| 57 | ID/RX/TT/AG | 37.5 | 1 | 0.26 |
| 58 | DD/RX/GT/GG | 37.5 | 1 | 0.26 |
| 59 | II/RR/GT/GG | 37.5 | 0 | 0 |
| 60 | II/RR/TT/AG | 37.5 | 0 | 0 |
| 61 | II/RX/TT/GG | 37.5 | 0 | 0 |
| 62 | ID/XX/TT/GG | 37.5 | 0 | 0 |
| 63 | DD/RX/GG/GG | 37.5 | 0 | 0 |
| 64 | DD/RX/GT/AG | 37.5 | 0 | 0 |
| 65 | DD/RX/TT/AA | 37.5 | 0 | 0 |
| 66 | DD/XX/GT/GG | 37.5 | 0 | 0 |
| 67 | DD/XX/TT/AG | 37.5 | 0 | 0 |
| 68 | ID/RR/GT/GG | 25 | 1 | 0.26 |
| 69 | DD/RR/GG/GG | 25 | 1 | 0.26 |
| 70 | DD/RR/GT/AG | 25 | 1 | 0.26 |
| 71 | DD/RX/TT/AG | 25 | 1 | 0.26 |
| 72 | II/RR/TT/GG | 25 | 0 | 0 |
| 73 | ID/RR/TT/AG | 25 | 0 | 0 |
| 74 | ID/RX/TT/GG | 25 | 0 | 0 |
| 75 | DD/RR/TT/AA | 25 | 0 | 0 |
| 76 | DD/XX/TT/GG | 25 | 0 | 0 |
| 77 | ID/RR/TT/GG | 12.5 | 0 | 0 |
| 78 | DD/RR/GT/GG | 12.5 | 0 | 0 |
| 79 | DD/RR/TT/AG | 12.5 | 0 | 0 |
| 80 | DD/RX/TT/GG | 12.5 | 0 | 0 |
| 81 | DD/RR/TT/GG | 0 | 0 | 0 |
