# Supplementary material for: Polygenic study of endurance-associated genetic markers ACE I/D, ACTN3 Arg(R)577Ter(X), CKMM A/G NcoI and eNOS Glu(G)298Asp(T) in male Gorkha soldiers
Source: Sports Med Open. 2017 Apr 26;3:17. doi: 10.1186/s40798-017-0085-0 (PMC5405041; doi:10.1186/s40798-017-0085-0)
Supplement: Supplementary file 7 — Comparison of physiological characteristics and VO2max in male Gorkha soldiers grouped according to total genotype score (TGS). (DOCX 32 kb) [file 40798_2017_85_MOESM7_ESM.docx]

**Polygenic study of endurance associated genetic markers *ACE I/D, ACTN3 Arg(R)577Ter(X)*, *CKMM A/G NcoI* and *eNOS Glu(G)298Asp(T)* in male Gorkha soldiers**

Journal Name: Sports Medicine

SeemaMalhotra, KiranPreet, ArvindTomar*, ShwetaRawat, Sayar Singh, Inderjeet Singh, L. Robert Varte, TirthankarChatterjee, M.S Pal and Soma Sarkar†

Defence Institute of Physiology and Allied Sciences (DIPAS), Ministry of Defence. Government of India, Lucknow Road, Delhi 110054.*Defence Research and Development Establishment (DRDE). Ministry of Defence, Government of India, Jhansi Road, Gwalior 474002, Madhya Pradesh.

†**CORRESPONDING AUTHOR:**

email: [soma_sarkar2000@yahoo.com](mailto:soma_sarkar2000@yahoo.com)

Table S7: Comparison of Physiological characteristics and VO_2max_ in male Gorkha soldiers grouped according to Total Genotype Score (TGS).

**a) Mean b) Oneway**

| **Report** | | | | | | | | | |
| --- | --- | --- | --- | --- | --- | --- | --- | --- | --- |
| TGS | | AGE | HEIGHT | WEIGHT | BMI | SYSBP | DYSBP | HR | VO2 |
| 1.00 | Mean | 30.2667 | 164.7267 | 62.9067 | 23.2000 | 121.9091 | 75.3636 | 72.0000 | 50.4280 |
|  | N | 15 | 15 | 15 | 15 | 11 | 11 | 11 | 5 |
|  | Std. Deviation | 9.19213 | 5.49070 | 5.70707 | 2.41838 | 13.12596 | 9.50024 | 10.55462 | 7.36269 |
| 2.00 | Mean | 29.2667 | 165.5100 | 64.4550 | 23.4850 | 121.9677 | 75.4194 | 68.9032 | 48.0708 |
|  | N | 60 | 60 | 60 | 60 | 31 | 31 | 31 | 13 |
|  | Std. Deviation | 7.75661 | 4.92753 | 7.31531 | 2.24302 | 9.10122 | 9.12788 | 13.33755 | 6.38792 |
| 3.00 | Mean | 28.6281 | 165.1149 | 64.3433 | 23.5742 | 121.3571 | 73.9048 | 69.0000 | 52.3915 |
|  | N | 121 | 121 | 120 | 120 | 84 | 84 | 84 | 41 |
|  | Std. Deviation | 7.60387 | 4.74594 | 8.19597 | 2.63466 | 12.50893 | 11.47613 | 9.94806 | 8.95651 |
| 4.00 | Mean | 28.9298 | 165.9193 | 65.3000 | 23.5254 | 120.0000 | 73.7922 | 68.5974 | 50.7893 |
|  | N | 114 | 114 | 114 | 114 | 77 | 77 | 77 | 41 |
|  | Std. Deviation | 7.91964 | 4.71112 | 7.22396 | 3.29633 | 12.38633 | 8.77247 | 11.80127 | 7.93563 |
| 5.00 | Mean | 31.9286 | 164.4333 | 63.5310 | 23.4762 | 121.6818 | 73.4545 | 75.3182 | 50.2369 |
|  | N | 42 | 42 | 42 | 42 | 22 | 22 | 22 | 16 |
|  | Std. Deviation | 8.24146 | 5.80658 | 8.08089 | 2.57510 | 13.87490 | 8.98002 | 19.91503 | 6.68106 |
| 6.00 | Mean | 26.1111 | 166.6556 | 63.3889 | 22.9222 | 122.5000 | 68.0833 | 64.5833 | 47.9500 |
|  | N | 18 | 18 | 18 | 18 | 12 | 12 | 12 | 4 |
|  | Std. Deviation | 8.50528 | 6.60341 | 5.81902 | 1.89692 | 11.43758 | 10.48339 | 11.10658 | 9.10147 |
| 7.00 | Mean | 34.0000 | 168.2750 | 69.6500 | 24.6000 |  |  |  | 48.5000 |
|  | N | 4 | 4 | 4 | 4 |  |  |  | 2 |
|  | Std. Deviation | 6.37704 | 3.51982 | 4.98364 | .98995 |  |  |  | 2.12132 |
| Total | Mean | 29.1952 | 165.4393 | 64.5153 | 23.4984 | 121.1097 | 73.7975 | 69.3586 | 50.8202 |
|  | N | 374 | 374 | 373 | 373 | 237 | 237 | 237 | 122 |
|  | Std. Deviation | 7.94363 | 5.01326 | 7.53366 | 2.73446 | 12.09026 | 9.99752 | 12.37283 | 7.92853 |

| **ANOVA** | | | | | | |
| --- | --- | --- | --- | --- | --- | --- |
|  | | Sum of Squares | df | Mean Square | F | Sig. |
| AGE | Between Groups | 641.818 | 6 | 106.970 | 1.715 | .116 |
|  | Within Groups | 22894.933 | 367 | 62.384 |  |  |
|  | Total | 23536.751 | 373 |  |  |  |
| HEIGHT | Between Groups | 148.213 | 6 | 24.702 | .983 | .437 |
|  | Within Groups | 9226.319 | 367 | 25.140 |  |  |
|  | Total | 9374.532 | 373 |  |  |  |
| WEIGHT | Between Groups | 281.773 | 6 | 46.962 | .825 | .551 |
|  | Within Groups | 20831.450 | 366 | 56.917 |  |  |
|  | Total | 21113.223 | 372 |  |  |  |
| BMI | Between Groups | 12.969 | 6 | 2.162 | .286 | .944 |
|  | Within Groups | 2768.570 | 366 | 7.564 |  |  |
|  | Total | 2781.539 | 372 |  |  |  |
| SYSBP | Between Groups | 160.212 | 5 | 32.042 | .216 | .956 |
|  | Within Groups | 34336.935 | 231 | 148.645 |  |  |
|  | Total | 34497.148 | 236 |  |  |  |
| VO_2max_ | Between Groups | 249.467 | 6 | 41.578 | .650 | .690 |
|  | Within Groups | 7356.794 | 115 | 63.972 |  |  |
|  | Total | 7606.261 | 121 |  |  |  |
| DYSBP | Between Groups | 503.900 | 5 | 100.780 | 1.008 | .413 |
|  | Within Groups | 23084.378 | 231 | 99.932 |  |  |
|  | Total | 23588.278 | 236 |  |  |  |
| HR | Between Groups | 1193.596 | 5 | 238.719 | 1.578 | .167 |
|  | Within Groups | 34934.919 | 231 | 151.233 |  |  |
|  | Total | 36128.515 | 236 |  |  |  |

1. Report of Mean; b) ANOVA;

Group Details: Group 1 with TGS 100.0; Group 2 with TGS: 87.5; Group 3 with TGS 75; Group 4 with TGS 62.5; Group 5 with TGS 50.0; Group 6 with TGS 37.5; Group 7 with TGS 25.

1. **Post hoc Tukey’s test**

**___________________________________________________________________**

95% Confidence

Interval

________________

Dependent (I) TGS (J) TGS Mean Std Sig* Lower Upper

Variable difference Error Bound Bound

__________________________________________________________________________

Age 1.00 7.00 -3.73 4.44 0.981 -16.91 9.44

Height 1.00 7.00 -3.54 2.82 0.871 -11.91 4.81

Weight 1.00 7.00 -6.74 4.24 0.690 -19.33 5.84

BMI 1.00 7.00 -1.40 1.54 0.972 -5.98 3.18

VO_2max_ 1.00 7.00 1.92 6.69 1.00 -18.15 22.01

___________________________________________________________________________

TGS 1 represent 100 score and 7 represent 25 score. *The mean difference is significant at the 0.05 level.
